# Miscellany

**Published:** 1909-06

**Authors:** 


### MISCELLANY.

---

A BOARD of commissioned medical officers will be convened to meet at the Bureau of Public Health and Marine-Hospital Service, 3 B street SE., Washington, D. C., Monday, June 14, 1909, at 10 o'clock a. m., for the purpose of examining candidates for admission to the grade of assistant surgeon in the Public Health and Marine-Hospital Service.

Candidates must be between 22 and 30 years of age, graduates of a reputable medical college, and must furnish testimonials from responsible persons as to their professional and moral character. For further information, or for invitation to appear before the board of examiners, address "Surgeon-General, Public Health and Marine-Hospital Service, Washington, D. C."

